# Socioemotional Factors and Cardiovascular Risk: What Is the Relationship in Brazilian Older Adults?

**DOI:** 10.1093/geroni/igad078

**Published:** 2023-07-26

**Authors:** Vanessa Pereira Corrêa, Cesar Messias de Oliveira, Danielle Soares Rocha Vieira, Carlos Alberto Severo Garcia, Ione Jayce Ceola Schneider

**Affiliations:** Department of Public Health, Postgraduate Program in Collective Health, Federal University of Santa Catarina, Florianópolis, Santa Catarina, Brazil; Department of Epidemiology and Public Health, University College London, London, UK; Department of Health Sciences, Postgraduate Program in Rehabilitation Sciences, Federal University of Santa Catarina, Araranguá, Santa Catarina, Brazil; Department of Health Sciences, Postgraduate Program in Rehabilitation Sciences, Federal University of Santa Catarina, Araranguá, Santa Catarina, Brazil; Department of Health Sciences, Postgraduate Program in Rehabilitation Sciences, Federal University of Santa Catarina, Araranguá, Santa Catarina, Brazil

**Keywords:** Cardiovascular diseases, Social interaction, Social support

## Abstract

**Background and Objectives:**

Cardiovascular risk is composed of several modifiable factors that cannot be explained only at the individual level. The aim of this study was to investigate the association between socioemotional factors and cardiovascular risk in older adults.

**Research Design and Methods:**

A cross-sectional study with data from The Brazilian Longitudinal Study of Aging (Estudo Longitudinal de Saúde dos Idosos Brasileiros, ELSI-Brazil), population based with data collected between 2015 and 2016. Cardiovascular risk—the study outcome—was assessed using the WHO/ISH Cardiovascular Risk Prediction Charts. The exposure variables were perceived social support from those who would receive help in situations and productive and leisurely social participation carried out in the last 12 months. We used crude and adjusted logistic regression for socioeconomic conditions, health conditions, and lifestyle habits to estimate odds ratio (OR) and 95% confidence intervals (CIs) for the occurrence of the outcome.

**Results:**

There were 6,005 participants between 50 and 74 years old with complete data. Of these, 18.7% (95% CI: 16.9–20.6) had high cardiovascular risk. Being in the highest tertile of greater social participation is associated with a lower prevalence of high cardiovascular risk (OR: 0.69; 95% CI: 0.50–0.95), adjusted for all variables, when compared to the lowest tertile. Furthermore, the absence of perceived social support is associated with a higher prevalence in different models. Perceived social support from close family members (son/daughter, son-in-law, and daughter-in-law) for material issues is associated with a higher prevalence, whereas having support from friends for affective resources is associated with a lower prevalence of high cardiovascular risk.

**Discussion and Implications:**

Socioemotional factors such as lack of perceived social support and social participation were significantly associated with cardiovascular risk. This suggested that the development of strategies aimed at reducing cardiovascular risk during aging needs to consider socioemotional factors and social relationships.

**Translational Significance:** Cardiovascular disease is a public health problem responsible for economic expenses and social issues. Understanding that there is a relationship between psychosocial factors and cardiovascular risk allows the planning of coping policies that include collective strategies and emphasis on contextual factors. The results of the study allow the planning of public policies and intersectoral programs for healthy and active aging from a perspective of social relations, as well as contributing to an international agenda for the management of cardiovascular risk.

In 2022, it is estimated that older adults represented more than 14.6% of the Brazilian population (IBGE, 2022). However, advances in living and health conditions have not kept up with the increase in life expectancy in low- and middle-income countries, leading to an increase in multimorbidity, especially in chronic noncommunicable diseases, which are responsible for severe degrees of disability and burden on the health and social security system (Miranda et al., 2016). In Brazil, cardiovascular disease affects about 14 million people and leads approximately 400,000 to death (SBC, 2022).

The association between cardiovascular disease and aging is well known. Brazilian longitudinal data show an increase in the prevalence rates of cardiovascular diseases over the years, as well as their association with increasing age. Physiological changes inherent to aging, associated with inappropriate lifestyle, can lead to the formation of atherosclerotic plaque. These are responsible for most cardiovascular outcomes (Alebiosu, 2001; Kita, 1998; Massa et al., 2019; Sniderman & Furberg, 2008). It is a multifactorial disease, and among risk factors are alcohol consumption, overweight, diabetes mellitus, systemic arterial hypertension, physical inactivity, age, and sex at birth. Except for age and sex at birth, the other factors are modifiable and mediate among themselves (Précoma et al., 2019).

Risk factors for cardiovascular disease can be quantified with risk scores that allow for prevention, prognostic analysis, and appropriate therapeutic decision. According to the literature, the definition of cardiovascular risk is the probability that the individual has of developing a cardiovascular outcome in each period (Kaptoge et al., 2019). This is the most effective way to prevent this condition because it allows early quantitative identification of fatal or nonfatal events, which is essential to find the appropriate therapeutic goal (OPAS, 2022). This stratification makes it possible and essential to identify asymptomatic individuals who are predisposed to cardiovascular disease. Although there are several instruments for stratifying cardiovascular risk (Nordet et al. 2013; OPAS, 2022; Précoma et al. 2019; Santos et al. 2014), most of them only consider biological risk factors, and this does not reflect all the determinants of cardiovascular diseases. Among the ways to understand the health-disease process, the continuum of care and health equity are essentials to cover the individual’s living space to understand the dynamics of social relations and psychosocial determinants (Rocha, 2018; Santos et al., 2009). The presence or absence of social support from social relationships influences coping strategies for stressful events. Thus, social relationships may be related to both the onset of the disease and coping with the condition itself (World Health Organization, 2005). Social–emotional factors, especially expanded relationships (participation) and assistance (support), have proven relationship with clinical outcomes, but there are still gaps in relation to cardiovascular risk (Rocha, 2018).

Studies point out that several professionals tend not to quantify this risk, producing an underestimation or overestimation of the individual’s predisposition to cardiovascular disease over the years (Kaptoge et al., 2019). Moreover, few studies address the use of these instruments in the Brazilian older adult population (Pimenta & Caldeira, 2014; Santos et al., 2014), with no research investigating the relationship between cardiovascular risk and biopsychosocial determinants. Brazil is part of the Hearts in the Americas initiative that is led by Pan American Health Organization in Latin America and provides for the implementation of the risk management model based on the score used in the present study (OPAS, 2022).

Given this, to understand how social relationships are related to cardiovascular risk through socioemotional factors—which consider social participation and aspects of social support—this study aimed to investigate the association between social–emotional factors and cardiovascular risk in older adults.

## Method

This is a cross-sectional study with data from The Brazilian Longitudinal Study of Aging (ELSI-Brazil), a longitudinal, population-based study, representative of the noninstitutionalized Brazilian population with 50 years of age or more. This study is an initiative coordinated by the Oswaldo Cruz Foundation—Minas Gerais and the Federal University of Minas Gerais. The baseline survey was carried out between 2015 and 2016, in 70 municipalities in the 5 Brazilian regions (Lima-Costa et al., 2022).

To ensure the sample represents urban and rural areas of municipalities of all sizes, the sample was recruited from a design with selection stages, combining stratification of primary sampling units (municipalities), census tracts, and households. Detailed information on design, recruitment methods, and covered topics are available in the study by Lima-Costa et al. (2023). The ELSI-Brazil project was approved by the Ethics Committee of Oswaldo Cruz Foundation (FIOCRUZ), Minas Gerais state (CAAE: 34649814.3.0000.5091). All participants signed separate informed consent forms prior to participating in the study (Lima-Costa et al., 2023).

In the present study, ELSI-Brazil participants with complete information were older adults between 50 and 74 years old. Individuals with 50 years of age were included because the changes inherent to aging start from the fifth decade of life, especially in low- and middle-income countries.

The outcome variable, cardiovascular risk, was calculated using the WHO/ISH Cardiovascular Risk Prediction Charts “Tropical Latin America” risk score only with nonlaboratory data, which provides a prediction graph and indicates the 10-year risk of a fatal or nonfatal cardiovascular event (acute myocardial infarction or stroke). The chart used can be found on page 50 of this link: https://apps.who.int/iris/bitstream/handle/10665/333221/9789240001367-eng.pdf). The used criteria are age (50–54, 55–59, 60–64, 65–69, 70–74 years old), sex at birth (female, male), systolic blood pressure (<120, 120–139, 140–159, 160–179, ≥180 mmHg), smoking (nonsmoker, former smoker more than12 months, smoker), and body mass index (<20, 20–24.9, 25–29.9, 30–34.9, ≥35 kg/m^2^; Kaptoge et al., 2019). The cardiovascular risk was categorized as low (<10%) and high (≥10%).

The exposure variables were social participation, obtained from 12 dichotomous questions (yes, no) about productive and leisure activities in the previous 12 months. The final score was categorized into tertile: lower (0–3), intermediate (4–6), and higher (7 or more). Perceived social support includes the sources of support for affective and instrumental resources, in the informal relationship network. The following situations were presented: (1) help in case of illness (diseases); (2) having someone to confide/trust in (confidence); (3) go shopping (shopping); and (4) lend money or objects (financial). For each situation, the participant should answer who would be present to help, with the response options: spouse or partner, child, daughter-in-law or son-in-law (close relatives), other relatives (distal relatives), others (friends, housekeeper, other paid employee, neighbors), and no one (Lima-Costa et al., 2019, 2023).

The adjustment variables were the social–structural: race/ethnicity (White, Black, Brown, Indigenous, Yellow)—classified according to the definitions proposed by the Brazilian Institute of Geography and Statistics (IBGE, 2023), education (no formal education, 1–4 years, 5–8 years, 9–11 years, ≥12 years), per capita income in tertiles and marital status (with a partner, without a partner); social–behavioral: physical activity (insufficiently active, active; Matsudo et al., 2012; World Health Organization, 2011), consumption of fruits and vegetables (adequate, inappropriate; Amine et al., 2003) and alcohol consumption (risky consumption, light/moderate, none; Sobell & Sobell, 1995); and health conditions: self-perceived health (positive, negative), noncommunicable chronic diseases (none, 1–2, ≥2; Brasil, 2020), dependence on basic activities of daily living (0, 1, or ≥2 activities; Brasil, 2020) and, cognitive function—time orientation (all right, at least one incorrect), memory (tertile), verbal fluency (tertile; Lourenço & Veras, 2006), and depressive symptoms (≤3 or ≥4; Batistoni et al., 2010; Karim et al., 2015; Turvey et al., 1999). It should be noted that the analyses were not adjusted for age, sex at birth, systolic blood pressure, smoking, and body mass index because they were variables in the composition of the cardiovascular risk score and were previously adjusted for the calculation of the outcome variable.

A statistical analysis of complete cases was performed. Descriptive analysis used absolute and relative frequencies of all study variables, with their respective confidence intervals (95% CI). To estimate the prevalence of the outcome occurrence, and the 95% CI, according to the other variables, a bivariate analysis with the test was used.χ². Analyses to estimate the chance of occurrence of the outcome according to the main exposures (financial support, confidence support, shopping support, diseases, and social participation) were performed using crude and adjusted logistic regression, with the odds ratio (OR) estimation with respective 95% CI. Adjusted analyses for each exposure were performed in blocks, according to the following models and variables: Model 1—social–structural (race/ethnicity, education, and income per capita); Model 2—health conditions (self-perceived health, chronic diseases, activities of daily living, cognitive function, and memory); Model 3—social–behavioral (physical activity, consumption of fruits and vegetables, and alcohol consumption) and Model 4—all variables (Model 1, Model 2, and Model 3). All analyses considered the sample weights using *svy* command and were performed using the statistical package *Stata SE* version 16 (StataCorp, 2019; Statistical Software: Release 16. College Station, TX: StataCorp LP).

In this study, we followed the “Strengthening the Reporting of Observational Studies in Epidemiology” (STROBE) guidelines (von Elm et al., 2008).

## Results

**Figure 1.**
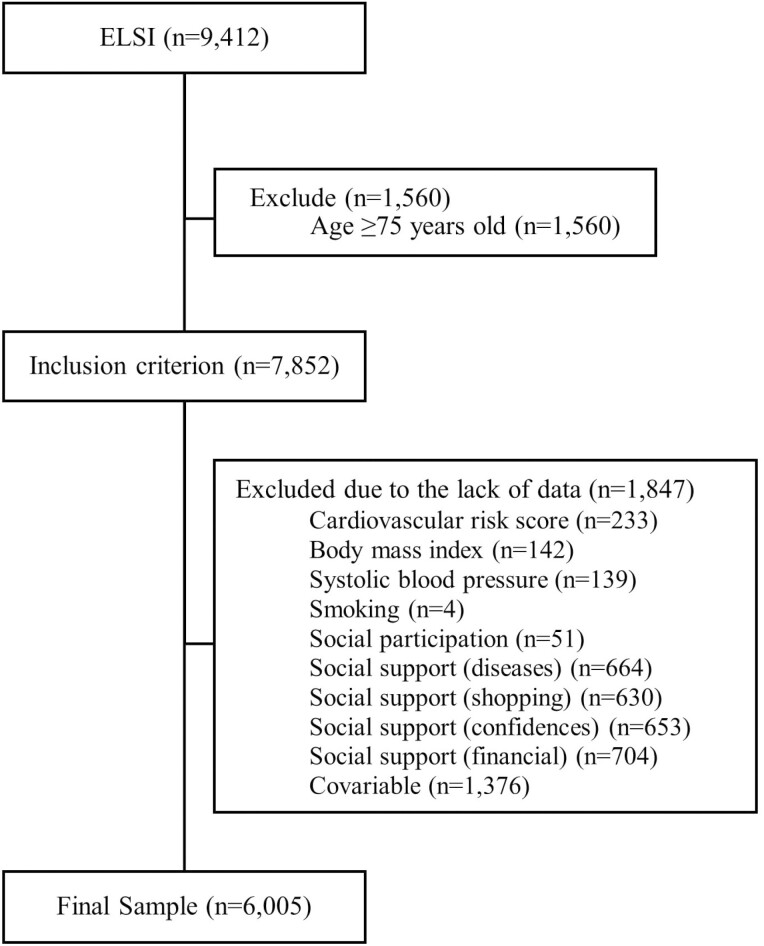
Sample selection flowchart considering the inclusion criterion of this study, ELSI-Brazil, 2015–2016. ELSI = The Brazilian Longitudinal Study of Aging.

6,005 participants between 50 and 74 years of age were included. The sample selection is shown in Figure 1. There is no evidence of differential losses based on a wide range of observed characteristics (Supplementary Table 1), despite the exclusion of 1,847 individuals from the analyses due to some missing information. The distribution of percentages within the variable categories was maintained.

Sample characterization can be seen in Table 1. Among the participants, most were between 50 and 59 years old (57.4%), female (52.2%), with a partner (68.5%), with low education (35.0%), highest income tertile (37.5%), and brown race/ethnicity (46.1%). The majority’s self-perception of health was positive (90.0%), they had no difficulty in ADL (87.9%), they were physically active (71.5%), they reported never smoking (44.3%), never drinking alcohol (78.4%), and had inadequate consumption of fruits and vegetables (91.3%). In addition, most have prehypertension stage (34.9%), overweight (40.8%), reported at least one noncommunicable chronic disease (33.9%), with good temporal orientation, verbal fluency (38.5%) and memory (40.7%), and ≤3 depressive symptoms (66.9%; Table 1).

**Table 1:** Descriptive Analysis of Participants’ Characteristics and Prevalence of High Cardiovascular Risk (≥10%), ELSI-Brazil, 2015–2016

Variable	n	% (95% CI)	CVR ≥10% % (95%CI)	p value^a^
Cardiovascular risk				
<10%	4,797	81.3 (79.4; 83.1)		
≥10%	1,208	18.7 (16.9; 20.6)		
Age group				<0.0001
50–59 years old	3,161	57.4 (53.4; 61.3)	4.4 (3.7–563)	
60–69 years old	2,143	33.4 (30.7; 36.2)	27.5 (25.2–30.0)	
70–74 years old	701	9.2 (7.7; 10.8)	75.6 (71.6–79.2)	
Sex at birth				<0.0001
Female	3,300	52.2 (48.8; 55.6)	10.4 (9.0–11.9)	
Male	2,705	47.8 (44.4; 51.2)	27.7 (24.1–31.7)	
Body mass index				0.0370
Normal	1,631	27.0 (25.7; 28.4)	21.0 (18.4–23.8)	
Underweight	112	1.8 (1.4; 2.3)	25.6 (16.9–36.7)	
Overweight	2,446	40.8 (39.3; 42.3)	17.4 (15.1–20.0)	
Obese	1,816	30.3 (28.8; 31.9)	17.9 (15.6–20.5)	
Systolic blood pressure				<0.0001
Normal	1,645	27.0 (25.3; 28.7)	3.9 (268–5.4)	
Prehypertension	2,308	38.1 (36.7; 39.6)	9.2 (7.3–11.4)	
Hypertension	2,052	34.9 (32.8; 36.9)	40.5 (36.7–44.4)	
Smoking				<0.0001
Never smoked	2,651	44.3 (42.4; 46.2)	10.7 (9.13–12.6)	
Former smoker	2,247	37.5 (35.2; 39.7)	19.6 (17.2–22.2)	
Smoker	1,107	18.3 (16.6; 20.0)	36.0 (32.6–39.4)	
Race/ethnicity				0.4591
White	2,290	41.4 (36.2; 46.7)	18.4 (15.6–21.5)	
Black	588	9.6 (8.0; 11.3)	2.0 (1.6–2.5)	
Brown	2,921	46.1 (41.9; 50.4)	18.2 (16.4–20.2)	
Yellow	56	0.9 (0.7; 1.3)	22.7 (13.1–36.3)	
Indigenous	150	1.9 (1.3; 2.7)	24.8 (17.5–33.9)	
Marital status				0.0002
Single	671	11.1 (9.6; 12.8)	14.3 (10.7–18.8)	
Married/common-law marriage/living together	3,783	68.5 (65.9; 71.0)	18.7 (16.5–21.1)	
Divorced or separated	768	10.8 (9.9; 11.9)	16.4 (13.4–19.9)	
Widower	783	9.5 (0.8; 10.9)	26.4 (23.0–30.1)	
Education				<0.0001
Never studied	694	9.4 (7.5; 11.6)	29.5 (26.2–33.1)	
1–4 years	2,168	35.0 (32.5; 37.6)	23.1 (20.6–25.9)	
5–8 years	1,359	23.7 (21.8; 25.7)	16.7 (13.8–20.2)	
9–11 years old	1,371	24.5 (22.7; 26.5)	12.0 (10.0–14.3)	
12 years or more	413	7.3 (6.1; 8.7)	1.2 (0.9–16.6)	
Income				0.0535
Highest tertile	2,104	37.5 (33.9; 41.3)	19.1 (16.9–21.5)	
Second tertile	1,876	31.1 (29.5; 32.7)	20.1 (17.5–23.1)	
Lowest tertile	2,025	31.4 (27.8; 35.1)	16.7 (14.6–19.1)	
Self-perception of health				0.9302
Positive	5,353	90.0 (88.5; 91.2)	1.9 (1.6–2.0)	
Negative	652	10.0 (8.8; 11.4)	18.8 (15.5–22.6)	
Chronic noncommunicable diseases				<0.0001
None	1,938	32.7 (30.9; 34.5)	14.4 (12.0–17.5)	
One condition	2,044	33.9 (32.1; 35.6)	19.0 (16.9–21.5)	
Two conditions or more	2,023	33.4 (31.1; 35.9)	22.3 (20.0–24.8)	
Activities of daily living				0.8403
No difficulty	5,246	87.9 (86.7; 89.0)	18.7 (16.8–20.8)	
One or more difficulties	759	12.0 (10.9; 13.3)	18.3 (15.2–21.9)	
Physical activity				0.0206
Active	4,282	71.5 (69.0; 73.8)	17.7 (15.9–19.6)	
Insufficiently active	1,723	28.5 (26.2; 30.9)	21.1 (18.1–24.5)	
Consumption of fruits and vegetables				0.0146
Adequate	472	8.6 (7.4; 10.0)	13.8 (10.2–18.3)	
Inadequate	5,533	91.3 (89.9; 92.6)	19.1 (17.3–21.1)	
Alcohol consumption				0.0001
Never	4,828	78.4 (75.6; 80.9)	17.5 (15.7–19.6)	
Light/moderate	559	10.8 (8.7; 13.3)	27.1 (22.2–32.6)	
Risk consumption	618	10.8 (9.7; 12.0)	18.5 (14.9–22.7)	
Time orientation				0.0001
All right	4,356	73.3 (71.5; 75.0)	17.4 (15.6–19.4)	
At least one incorrect	1,649	26.7 (25.0; 28.5)	22.1 (19.5–24.9)	
Memory				<0.0001
Lower tertile	2,135	33.5 (30.9; 36.2)	25.3 (22.6–28.3)	
Intermediate tertile	1,535	25.8 (24.3; 27.4)	16.7 (14.4–19.3)	
Top tertile	2,335	40.7 (38.5; 42.9)	14.4 (12.6–16.6)	
Verbal fluency test				0.0023
Lower tertile	2,055	32.1 (29.4; 35.0)	21.4 (19.0–24.1)	
Intermediate tertile	1,748	29.3 (27.8; 30.9)	19.0 (16.8–21.3)	
Top tertile	202	38.5 (35.6; 41.4)	16.2 (13.7–18.9)	
Depressive symptoms				<0.0001
≤3 symptoms	3,939	66.9 (64.9; 68.8)	20.4 (18.4–22.7)	
≥4 symptoms	2,066	33.1 (31.2; 35.1)	15.1 (13.1–17.5)	
Social participation				<0.0001
Lower tertile	1,736	27.1 (23.9; 30.6)	23.6 (20.9–26.6)	
Intermediate tertile	2,203	35.9 (34.1; 37.6)	19.3 (17.0–21.7)	
Top tertile	2,066	37.0 (33.0; 41.0)	14.5 (12.4–16.9)	
Support (diseases)				0.0072
Spouse or partner	1,655	29.0 (27.2; 30.8)	18.5 (16.0–21.3)	
Son/daughter/son-in-law/daughter-in-law	2,535	40.3 (38.2; 42.3)	20.9 (18.6–23.3)	
Another relative	940	16.8 (15.7; 17.9)	14.2 (11.4–17.6)	
Other	703	11.3 (10.0; 12.6)	18.0 (14.5–22.2)	
Nobody	172	2.6 (2.2; 3.2)	18.2 (12.3–26.2)	
Support (shopping)				0.0022
Spouse or partner	1,874	33.6 (31.1; 36.2)	15.4 (13.4–17.6)	
Son/ daughter/son-in-law/daughter-in-law	3,028	47.5 (45.2; 49.9)	20.7 (18.5–23.1)	
Another relative	683	12.1 (11.0; 13.2)	18.2 (14.1–23.2)	
Other	324	5.3 (4.7; 6.3)	20.5 (15.7–26.4)	
Nobody	96	1.5 (1.2; 1.8)	25.7 (17.4–36.3)	
Support (confidence)				0.0245
Spouse or partner	2,012	35.6 (33.1; 38.2)	19.9 (17.5–22.6)	
Son/daughter/son-in-law/daughter-in-law	2,024	30.8 (28.8; 32.9)	20.3 (18.0–22.7)	
Another relative	776	13.6 (12.4; 14.8)	14.2 (11.1–18.1)	
Other	742	12.8 (11.8; 13.8)	15.8 (12.4–20.0)	
Nobody	451	7.2 (6.3; 8.1)	19.2 (14.0–25.6)	
Support (financial)				<0.0001
Spouse or partner	943	16.5 (15.0; 18.1)	14.0 (12.1–16.2)	
Son/daughter/son-in-law/daughter-in-law	2,091	32.4 (30.5; 34.3)	22.5 (20.0–25.1)	
Another relative	1,228	22.1 (20.6; 23.7)	12.5 (10.1–15.4)	
Other	1,184	19.7 (18.2; 21.3)	20.3 (17.2–23.7)	
Nobody	559	9.2 (8.1; 10.6)	25.0 (19.6–31.3)	

*Notes*: CI = confidence interval; CVR = cardiovascular risk; ELSI = The Brazilian Longitudinal Study of Aging.

^a^ *p* Value <.0001 was considered significant.

Regarding the social participation score, most were in the highest tertile (37.0%). As for support in the network of informal relationships, most reported their children/sons-in-law/daughters-in-law as the main source of support when related to shopping (47.5%), diseases, or financial help (32.4%). Regarding confidences/trust, they reported the spouse as the main source of support (35.6%).

**Figure 2.**
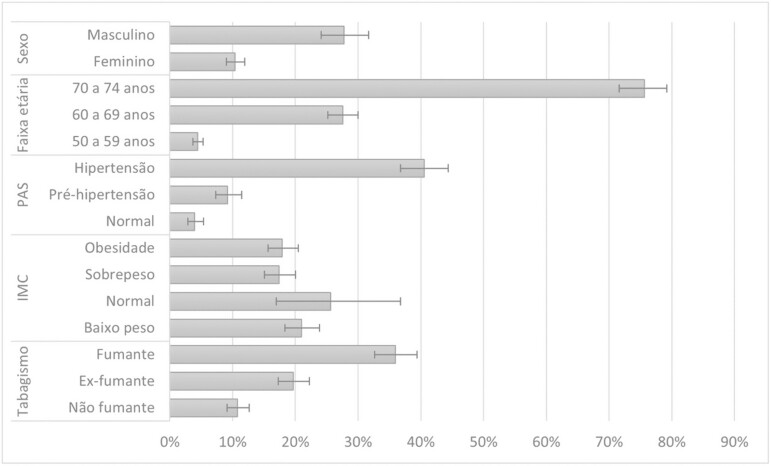
Prevalence of high cardiovascular risk in different characteristics of the cardiovascular risk score, ELSI-Brazil, 2015–2016. ELSI = The Brazilian Longitudinal Study of Aging.

Of the participants, 18.7% (95% CI: 16.9–20.6) had a cardiovascular risk greater than or equal to 10% (Table 1). Regarding the variables that make up the cardiovascular risk, the prevalence was higher in the group between 70 and 74 years old (75.6%), in males (27.7%), in those with low weight (25.6%), with systemic arterial hypertension (40.5%), and smokers (36.0%; Figure 2).

The prevalence of high cardiovascular risk was significantly higher in those of indigenous race/ethnicity (24.8%), widowed (26.8), who did not study (29.5%), intermediate income tertile (20.1%), with negative health self-perception (18.8%), with two or more noncommunicable chronic diseases (22.3%), without difficulty in activities of daily living (18.7%), insufficiently active (21.1%), who inadequately consumed fruits and vegetables (19.1%), with light/moderate alcohol consumption (27.1%), in the lowest tertile of memory (25.3%) and verbal fluency (21.4%) scores, and with ≥4 depressive symptoms (20.4%; Table 1).

Regarding social–emotional variables (Table 1), high cardiovascular risk was higher among those in the lowest tertile of social participation (23.6%), with support from a son/daughter/son-in-law/daughter-in-law in case of illness (20.9%), confidential (20.3%), without support for shopping (25.7%), and without financial support (25.0%).

In the multivariate analysis (Table 2), being in the upper tertile of social participation is associated with a lower prevalence of high cardiovascular risk, both in the crude analysis and adjusted by all models, by up to 48.0% (95% CI: 0.41–0.66). Being in the group categorized as a medium tertile of social participation was significant only in the crude analysis and when adjusted for lifestyle habits. Both were compared to the lower tertile of social participation.

**Table 2.** Crude and Adjusted Multivariate Analysis of Cardiovascular Risk, ELSI-Brazil, 2015–2016

Variables	Crude model	Model 1^a^	Model 2^a^	Model 3^a^	Model 4^a^
	OR (95% CI)	OR (95% CI)	OR (95% CI)	OR (95% CI)	OR (95% CI)
Social participation					
Lower tertile	1	1	1	1	1
Intermediate tertile	0.77 (0.64–0.92)	0.83 (0.68–1.00)	0.84 (0.68–1.03)	0.76 (0.62–0.92)	0.85 (0.69–1.06)
Top tertile	0.54 (0.44-0.68)	0.68 (0.51-0.89)	0.64 (0.48-0.84)	0.52 (0.41-0.66)	0.69 (0.50-0.95)
Support (disease)					
Spouse or partner	1	1	1	1	1
Son/daughter/son-in-law/daughter-in-law	1.16 (0.95–1.40)	0.99 (0.80–1.22)	1.12 (0.92–1.37)	1.21 (0.99–1.47)	1.05 (0.84–1.30)
Another relative	0.72 (0.58–0.91)	0.79 (0.62–0.99)	0.75 (0.60–0.94)	0.72 (0.57–0.91)	0.80 (0.63–1.01)
Other	0.96 (0.72–1.28)	0.86 (0.63–1.17)	0.93 (0.70–1.23)	0.98 (0.73–1.31)	0.87 (0.63–1.20)
Nobody	0.98 (0.62–1.54)	0.89 (0.53–1.48)	1.07 (0.68–1.68)	0.99 (0.63–1.57)	1.01 (0.61–1.69)
Support (shopping)					
Spouse or partner	1	1	1	1	1
Son/daughter/son-in-law/daughter-in-law	1.43 (1.21–1.70)	1.33 (1.09–1.62)	1.38 (1.15–1.65)	1.48 (1.24–1.76)	1.37 (1.11–1.69)
Another relative	1.22 (0.89–1.67)	1.43 (1.02–2.02)	1.24 (0.89–1.72)	1.24 (0.90–1.69)	1.46 (1.02–2.09)
Other	1.42 (1.01–2.00)	1.46 (0.97–2.19)	1.48 (1.03–2.13)	1.43 (1.01–2.01)	1.55 (1.02–2.09)
Nobody	1.90 (1.10–3.27)	1.97 (1.09–3.56)	2.13 (1.17–3.86)	1.91 (1.12–3.25)	2.28 (1.25–4.15)
Support (confidence)					
Spouse or partner	1	1	1	1	1
Son/daughter/ son-in-law/daughter-in-law	1.02 (0.85–1.21)	0.84 (0.68–1.02)	0.96 (0.81–1.15)	1.06 (0.89–1.27)	0.89 (0.72–1.09)
Another relative	0.66 (0.50–0.88)	0.68 (0.51–0.92)	0.70 (0.53–0.93)	0.68 (0.51–0.91)	0.75 (0.56–1.00)
Other	0.75 (0.56–1.01)	0.76 (0.55–1.06)	0.80 (0.60–1.08)	0.78 (0.59–1.05)	0.84 (0.60–1.17)
Nobody	0.95 (0.63–1.42)	0.95 (0.61–1.47)	1.03 (0.67–1.58)	0.94 (0.63–1.40)	1.02 (0.65–1.61)
Support (financial)					
Spouse or partner	1	1	1	1	1
Son/daughter/son-in-law/daughter-in-law	1.77 (1.43–2.19)	1.63 (1.30–2.05)	1.72 (1.38–2.14)	1.78 (1.44–2.21)	1.65 (1.31–2.07)
Another relative	0.87 (0.64–1.18)	1.04 (0.77–1.40)	0.93 (0.69–1.26)	0.83 (0.61–1.13)	1.00 (0.73–1.37)
Other	1.55 (1.18–2.03)	1.56 (1.19–2.06)	1.61 (0.69–2.11)	1.50 (1.13–1.98)	1.55 (1.16–2.07)
Nobody	2.03 (1.41-2.92)	2.02 (1.36–2.99)	2.09 (1.42–3.08)	1.97 (1.36–2.83)	2.04 (1.36–3.05)

*Notes*: ADL = activity of daily living; CI = confidence interval; ELSI = The Brazilian Longitudinal Study of Aging; OR = odds ratio.

^a^Model 1 (race/color, education, and income); Model 2 (self-perceived health, chronic diseases, ADL, cognitive function and memory); Model 3 (physical activity, consumption of fruits and vegetables and alcohol consumption), and Model 4 (Model 1, Model 2, and Model 3).

Regarding perceived social support, receiving support from other relatives in cases of disease is associated with a lower prevalence of high risk in both the crude and adjusted analyses by up to 28% (95% CI: 0.57–0.91) compared to receiving support from the spouse, except when adjusted for all variables. In the case of support for confidence, the result was similar: receiving it from other relatives is associated with a lower prevalence of high risk—even when adjusted to all models—by up to 32% (95% CI: 0.51–0.91), except when adjusted for all variables (Table 2).

In the case of perceived social support for shopping, there was a significant association with the prevalence of high cardiovascular risk, adjusted for all variables, especially for receiving no support (OR: 2.28; 95% CI: 1.25–4.15), when comparing receiving support from a spouse. Receiving confidential support from another relative is associated with a lower prevalence of high cardiovascular risk, by up to 32%, except in the model adjusted for all variables, in relation to the spouse. Regarding financial support, except support from other relatives, it is significantly associated with the prevalence of high cardiovascular risk compared to receiving support from the spouse. Only support from others (nonfamily members) adjusted for health conditions did not remain significant (Table 2).

## Discussion

The present study identified prevalence of cardiovascular risk ≥10% in 18.7% of participants and was associated with social–emotional factors. Being in the upper tertile of social participation is associated with a prevalence of high cardiovascular risk as well as having support from friends for confidence. The absence of social support for material issues was associated with a prevalence of high cardiovascular risk. This is a relevant finding considering the scarcity of studies investigating this association and considering health determinants and health as a social process.

A study carried out in Malaysia, Mongolia, and Cambodia, developing countries, using score charts created by World Health Organization and the International Society of Hypertension, shows the high cardiovascular risk in 2.3%, 6.0%, and 1.3% of the participants, respectively. However, the study was carried out with individuals between 40 and 64 years old (*n* = 7,109), as the aging process starts around the fifth decade of life or before in countries with such inequities in health (Otgontuya et al., 2013). In Cuba, a country that does not have the development index listed by the International Monetary Fund, but has excellent development indices and a model health system, a study was carried out with the same score that indicates less than 10% of participants with high cardiovascular risk (Nordet et al., 2013), corroborating other findings (Mendis et al., 2011; Ndindjock et al., 2011; Otgontuya et al., 2013) and the findings of this study.

The association between social–emotional factors and high cardiovascular risk was the main finding of this study. The absence of perceived social support or from close family members, such as financial or shopping assistance, presented association with the prevalence of high cardiovascular risk, whereas greater social participation reduced it. Social–emotional factors correspond to affective resources—perceived social support—and the relationship, integration, and role that the individual plays in society, regardless of work—social participation(Pelcastre-Villafuerte et al., 2011). No studies were found that stratified the cardiovascular risk score/status associated with perceived social support and social participation; however, studies were identified that pointed to the relationship with cardiovascular risk factors, individually—for example, associated only with smoking or with age—or with cardiovascular disease (Fletcher & Bulpitt, 1992; Valtorta et al., 2018).

Social connections can influence different pathways, in coronary heart disease and stroke, such as adherence to treatment, adherence to appropriate habits, and effects on biological markers (Holt-Lunstad & Smith, 2016). The findings of the present study corroborate the hypothesis that the expansion of social relationships at the level of social involvement positively contributes to positive outcomes in the individual’s health.

Regarding diseases or confidence support, which correspond to the affective resource, receiving support from other relatives—other than close relatives, such as sons, daughters, daughters-in-law, and sons-in-law—reduced the prevalence of high cardiovascular risk compared to receiving support from the spouse. The main source of informal support during aging is family members (Pelcastre-Villafuerte et al., 2011). Social relationship networks decrease with aging; however, the older adults who have interpersonal relationships that provide emotional resources, such as support for confidences, tend to face stressful events more favorably (Neri & Vieira, 2013). On the other hand, loneliness can be a stress factor and increase cardiovascular risk through several mechanisms, such as increased cortisol concentrations, increased blood pressure, increased levels of inflammatory markers, as well as exacerbating other psychosocial and behavioral risk factors (Valtorta et al., 2018)

However, the purchase and financial support received from sons, daughters, daughters-in-law, and sons-in-law (i.e., those closest relatives) were associated with the prevalence of high cardiovascular disease when compared to the support received from the spouse. Studies pointing to this relationship were not found, but receiving instrumental support may reflect self-perception of functional incapacity, as shopping is considered a daily living activity, and receiving financial support may be related to individual productive capacity. There has been an evolution in the field of studies of the connections and/or social involvement and behaviors related to health conditions. Above all, it is essential to approach the influence of social–emotional aspects through population-based longitudinal studies to define appropriate strategies and subsidize the qualification of specific public policies for the comprehensive care of the older adult population, whether dependent or not (Minayo, 2019).

The groups that present a higher prevalence of high cardiovascular risk in this study are part of the cardiovascular risk factors, except for low weight. No associations were found to explain the high cardiovascular risk in underweight individuals; however, it may be related to inadequate diet, sarcopenia, nontransmissible chronic diseases, social isolation, depressive symptoms, and low purchasing power, among other factors related to the development of cardiovascular diseases.

In this study, more than two thirds were overweight or obese, had prehypertension stage, and had at least one chronic disease. Inadequate lifestyle is a proven risk factor for the onset of nontransmissible chronic diseases, and in the present study, most participants reported inadequate consumption of fruits, vegetables, and greens. According to the World Health Organization, most of these conditions could be avoided by maintaining adequate nutritional intake and regular physical activity (World Health Organization, 2011). Moreover, overweight and hypertension are also proven factors for increased cardiovascular risk, onset, and worse prognosis of cardiovascular disease. The presence of chronic nontransmissible diseases can cause increased cardiovascular risk by accelerating the loss of functional reserve in the affected organ, as well as systemic changes that lead to homeostatic imbalance (Kritsilis et al., 2018).

In the present study, most participants were between 50 and 59 years old, a hypothesis that could explain the lower percentage of cardiovascular risk when compared to other studies because cardiovascular risk increases with age. The highest prevalence of high cardiovascular risk was found in the age group between 70 and 74 years old, and several studies show the relationship between increasing age, factors, and risk itself (Fukutomi & Kario, 2010; Izzo et al., 2018).

Among the limitations of the study is the difficulty in establishing temporal causal relationships due to the cross-sectional design and use of observational data; however, the objective is to propose the use of the score in Primary Health Care that will also work with secondary data. In addition, it is worth mentioning the potential information bias, especially in relation to smoking information, which is part of the score and was self-reported, and the bias of the instrument, because the score used does not consider family history and the use of medications. These factors could increase the prevalence of high cardiovascular risk and demand caution in inferring these results. It is also important to clarify that the use of scores has some limitations of its own, for example, all nonsmoking women with systolic blood pressure <160 are classified in the lowest risk category, regardless of any other characteristics. On the other hand, 40- to 44-year-old smokers cannot be classified into any of the higher-risk categories in this model, whereas 70- to 74-year-old smokers cannot be classified as below-average risk in this model. Therefore, it is important to associate the use of the score with other social and clinical issues.

However, the results found can subsidize the creation of strategies to reduce inequities, supporting clinical practice in the management of cardiovascular risk through the approach of social–emotional factors, especially in relation to social support.

It highlights the need to consider spaces that promote social bonds, paths that have already been explored in the agenda of several countries, especially Brazil, where there are laws, public policies, and guides for healthy aging, not necessarily adequately funded or put into practice by health services. Despite the advances in public policies, programs, and services focused on aging, the approach to modifiable risk factors for the development of cardiovascular disease is mostly carried out only through physical activity and healthy eating.

Finally, it is necessary to understand the indispensable need for health care to increase the physical, cognitive, emotional, and social health autonomy of the older adult population, without leaving aside the different types of vulnerabilities existing in society. Thus, the promotion of formal social support networks and the awareness of informal social support networks can establish support points for the management of these factors, both in the State and in civil society, considering, finally, that social–emotional factors increase the prevalence of cardiovascular risk not only by physiological order, but also by acting as a stressor agent that makes it impossible to face the events that happen during the aging process.

Moreover, no significant difference was found between the high risk in using the scoring charts with and without cholesterol (Mendis et al., 2011), which shows that the choice of the instrument used in the present study is adequate, as well as it has applicability for low- and middle-income countries, contributing to the Hearts and Hearts in the Americas initiative. We suggest other studies of longitudinal design that address the relationship between the cardiovascular risk and exposure to social–emotional factors. In addition, further studies at different stages of aging are warranted to examine the impact of social–emotional factors on the increase in cardiovascular risk.

The study found a relationship between social–emotional factors and cardiovascular risk in aging. Both perceived social support in informal networks and social participation impacted the prevalence of cardiovascular risk. Interventions directed at healthy aging, with emphasis on social support and social participation, are one of the ways to address modifiable risk factors.

## Supplementary Material

igad078_suppl_Supplementary_Material

## Data Availability

The data of the present study can be found at http://elsi.cpqrr.fiocruz.br/.
