# Supplementary material for: Socioemotional Factors and Cardiovascular Risk: What Is the Relationship in Brazilian Older Adults?
Source: Innov Aging. 2023 Jul 26;7(7):igad078. doi: 10.1093/geroni/igad078 (PMC10506174; doi:10.1093/geroni/igad078)
Supplement: igad078_suppl_Supplementary_Material [file igad078_suppl_supplementary_material.docx]

**Online Supplementary Material**

Supplementary Table 1. Description of all cases, complete and excluded cases, ELSI-Brasil, 2015-2016.

| **Variable** | | **Inclusion criterion** |  | **Final sample** |
| --- | --- | --- | --- | --- |
|  |  | **%(IC95%)** |  | **%(IC95%)** |
| **Cardiovascular Risk** | |  |  |  |
|  | <10% | 80.6 (78.7;82.3) |  | 81.3 (79.4;83.1) |
|  | ≥10% | 19.4 (17.7;21.3) |  | 18.7 (16.9;20.6) |
| **Age group** | |  |  |  |
|  | 50 to 59 years old | 55.4 (51.7;59.1) |  | 57.4 (53.4;61.3) |
|  | 60 to 69 years old | 34.5 (32.0;37.1) |  | 33.4 (30.7;36.3) |
|  | 70 to 74 years old | 10.1 (8.7;11.7) |  | 9.2 (7.7;10.9) |
| **Sex at birth** | |  |  |  |
|  | Female | 52.9 (49.7;56.1) |  | 52.2 (48.8;55.6) |
|  | Male | 47.1 (43.9;50.3) |  | 47.8 (44.4;51.2) |
| **Body Mass Index** | |  |  |  |
|  | Normal | 27.8 (26.6;29.1) |  | 27.0 (25.7;28.4) |
|  | Underweight | 1.8 (1.5;2.3) |  | 1.9 (1.5;2.3) |
|  | Overweight | 39.9 (38.5;41.4) |  | 40.8 (39.3;42.3) |
|  | Obese | 30.4 (29.0;31.9) |  | 30.3 (28.8;31.9) |
| **Systolic Blood Pressure** | |  |  |  |
|  | Normal | 26.5 (25.0;28.1) |  | 27.0 (25.3;28.7) |
|  | Prehypertension | 37.8 (36.4;39.1) |  | 38.2 (36.7;39.7) |
|  | Hypertension | 35.7 (33.7;37.7) |  | 34.9 (32.9;37.0) |
| **Smoking** | |  |  |  |
|  | Never smoked | 44.6 (42.6;46.6) |  | 44.3 (42.4;46.2) |
|  | Former smoker | 36.8 (34.8;38.9) |  | 37.5 (35.3;39.7) |
|  | Smoker | 18.6 (16.9;20.4) |  | 18.3 (16.6;20.1) |
| **Race/ethnicity** | |  |  |  |
|  | White | 42.2 (36.8;47.9) |  | 41.4 (36.2;46.8) |
|  | Black | 9.7 (8.0;11.7) |  | 9.6 (8.1;11.4) |
|  | Brown | 45.2 (40.8;49.7) |  | 46.1 (41.9;50.4) |
|  | Yellow | 1.0 (0.7;1.3) |  | 1.0 (0.7;1.4) |
|  | Indigenous | 1.9 (1.3;2.7) |  | 1.9 (1.4;2.7) |
| **Marital status** | |  |  |  |
|  | Single | 12.3 (10.8;13.9) |  | 11.1 (9.7;12.8) |
|  | Married / Common-law marriage / Living together | 66.6 (63.8;69.3) |  | 68.6 (66.0;71.0) |
|  | Divorced or separated | 10.9 (10.0;12.0) |  | 10.9 (9.9;11.9) |
|  | Widower | 10.2 (8.9;11.7) |  | 9.5 (8.3;10.9) |
| **Education** | |  |  |  |
|  | Never studied | 7.0 (5.9;8.1) |  | 7.3 (6.2;8.7) |
|  | 1 to 4 years | 22.4 (20.5;24.4) |  | 24.6 (22.7;26.5) |
|  | 5 to 8 years | 23.4 (21.1;25.9) |  | 23.7 (21.8;25.7) |
|  | 9 to 11 years old | 36.3 (33.9;38.7) |  | 35.0 (32.5;37.6) |
|  | 12 years or more | 10.9 (9.0;13.2) |  | 9.4 (7.6;11.6) |
| **Income** | |  |  |  |
|  | Highest tertile | 35.3 (31.8;39.0) |  | 37.5 (33.9;41.3) |
|  | Second tertile | 31.6 (29.8;33.6) |  | 31.1 (29.6;32.7) |
|  | Lowest tertile | 33.1 (29.3;37.1) |  | 31.4 (27.9;35.1) |
| **Self-perception of health** | |  |  |  |
|  | Positive | 88.8 (87.4;90.0) |  | 90.0 (88.6;91.2) |
|  | Negative | 11.2 (10.0;12.6) |  | 10.0 (8.8;11.4) |
| **Chronic non-communicable diseases** | |  |  |  |
|  | None | 32.6 (30.9;34.4) |  | 32.7 (30.9;34.5) |
|  | One condition | 34.3 (33.0;35.7) |  | 33.9 (32.1;35.6) |
|  | Two conditions or more | 33.1 (31.1;35.1) |  | 33.5 (31.1;35.9) |
| **Activities of daily living** | |  |  |  |
|  | No difficulty | 86.0 (84.8;87.1) |  | 88.0 (86.7;89.1) |
|  | One or more difficulties | 14.0 (12.9;15.2) |  | 12.0 (10.9;13.3) |
| **Physical activity** | |  |  |  |
|  | Active | 69.2 (66.6;71.7) |  | 71.5 (69.1;73.8) |
|  | Insufficiently active | 30.8 (28.3;33.4) |  | 28.5 (26.2;30.9) |
| **Consumption of fruits and vegetables** | |  |  |  |
|  | Adequate | 8.4 (7.4;9.6) |  | 8.6 (7.4;10.1) |
|  | Inadequate | 91.6 (90.4;92.6) |  | 91.4 (90.0;92.6) |
| **Alcohol consumption** | |  |  |  |
|  | Never | 79.5 (77.0;81.8) |  | 78.4 (75.6;80.9) |
|  | Light/moderate | 10.5 (8.7;12.7) |  | 10.8 (8.7;13.4) |
|  | Risk consumption | 10.0 (9.0;11.0) |  | 10.8 (9.7;12.0) |
| **Time orientation** | |  |  |  |
|  | All right | 71.7 (69.7;73.5) |  | 73.3 (71.5;75.0) |
|  | At least one incorrect | 28.4 (26.5;30.3) |  | 26.7 (25.0;28.5) |
| **Memory** | |  |  |  |
|  | Lower tertile | 35.8 (33.2;38.6) |  | 33.5 (30.9;36.2) |
|  | Intermediate tertile | 25.7 (24.3;27.2) |  | 25.8 (24.3;27.4) |
|  | Top tertile | 38.5 (36.4;40.6) |  | 40.7 (38.5;42.9) |
| **Verbal fluency test** | |  |  |  |
|  | Lower tertile | 35.7 (32.8;38.7) |  | 32.2 (29.4;35.1) |
|  | Intermediate tertile | 28.7 (27.3;30.2) |  | 29.4 (27.8;30.9) |
|  | Top tertile | 35.6 (32.9;38.4) |  | 38.5 (35.6;41.4) |
| **Depressive symptoms** | |  |  |  |
|  | <=3 symptoms | 66.2 (64.4;68.0) |  | 66.9 (64.9;68.8) |
|  | >=4 symptoms | 33.8 (32.0;35.6) |  | 33.1 (31.2;35.1) |
| **Social Participation** | |  |  |  |
|  | Lower tertile | 30.3 (26.6;34.2) |  | 27.1 (24.0;30.6) |
|  | Intermediate tertile | 35.2 (33.5;36.9) |  | 35.9 (34.1;37.7) |
|  | Top tertile | 34.6 (30.5;38.9) |  | 37.0 (33.1;41.1) |
| **Support (diseases)** | |  |  |  |
|  | Spouse or partner | 28.1 (26.4;29.9) |  | 29.0 (27.2;30.8) |
|  | Son / Daughter / Son-in-law/ Daughter-in-law | 40.1 (38.2;42.1) |  | 40.3 (38.3;42.3) |
|  | Another relative | 17.6 (16.6;18.6) |  | 16.8 (15.8;17.9) |
|  | Other | 11.5 (10.3;12.9) |  | 11.3 (10.1;12.6) |
|  | Nobody | 2.7 (2.3;3.3) |  | 2.7 (2.2;3.2) |
| **Support (shopping)** | |  |  |  |
|  | Spouse or partner | 32.5 (30.1;35.0) |  | 33.6 (31.1;36.2) |
|  | Son / Daughter / Son-in-law/ Daughter-in-law | 47.4 (45.4;49.4) |  | 47.5 (45.2;49.9) |
|  | Another relative | 12.7 (11.7;13.7) |  | 12.1 (11.0;13.2) |
|  | Other | 5.7 (4.9;6.7) |  | 5.3 (4.5;6.3) |
|  | Nobody | 1.7 (1.4;2.1) |  | 1.5 (1.2;1.9) |
| **Support (confidence)** | |  |  |  |
|  | Spouse or partner | 33.9 (31.4;36.6) |  | 35.6 (33.1;38.2) |
|  | Son / Daughter / Son-in-law/ Daughter-in-law | 31.3 (29.3;33.4) |  | 30.8 (28.8;32.9) |
|  | Another relative | 14.0 (12.9;15.2) |  | 13.6 (12.5;14.8) |
|  | Other | 13.0 (12.2;13.9) |  | 12.8 (11.8;13.8) |
|  | Nobody | 7.8 (6.9;8.8) |  | 7.2 (6.3;8.2) |
| **Support (financial)** | |  |  |  |
|  | Spouse or partner | 15.8 (14.4;17.2) |  | 16.5 (15.0;18.1) |
|  | Son / Daughter / Son-in-law/ Daughter-in-law | 32.4 (30.5;34.4) |  | 32.4 (30.5;34.3) |
|  | Another relative | 22.1 (20.8;23.5) |  | 22.1 (20.6;23.7) |
|  | Other | 20.1 (18.6;21.6) |  | 19.7 (18.2;21.3) |
|  | Nobody | 9.7 (8.6;11.0) |  | 9.3 (8.1;10.6) |
